# Design Principles for Shared Digital Twins in Distributed Systems

**DOI:** 10.1007/s12599-022-00751-1

**Published:** 2022-04-15

**Authors:** Hendrik Haße, Hendrik van der Valk, Frederik Möller, Boris Otto

**Affiliations:** 1 Fraunhofer Institute for Software and Systems Engineering, ISST, Emil-Figge-Straße 91, 44227 Dortmund, Germany; 2 Industrial Information Management IIM, TU Dortmund University, Joseph-von-Fraunhofer-Straße 2-4, 44227 Dortmund, Germany

**Keywords:** Digital Twin, Design principles, Data sharing, Qualitative research

## Abstract

Digital Twins offer considerable potential for cross-company networks. Recent research primarily focuses on using Digital Twins within the limits of a single organization. However, Shared Digital Twins extend application boundaries to cross-company utilization through their ability to act as a hub to share data. This results in the need to consider additional design dimensions which help practitioners design Digital Twins tailored for inter-company use. The article addresses precisely that issue as it investigates how Shared Digital Twins should be designed to achieve business success. For this purpose, the article proposes a set of design principles for Shared Digital Twins stemming from a qualitative interview study with 18 industry experts. The interview study is the primary data source for formulating and evaluating the design principles.

## Introduction

Digital Twins currently attract considerable attention in both research and business practice (Tao et al. 2019b; Zhao et al. 2019a). On the one hand, this results in the steadily increasing number of publications on Digital Twins and, on the other hand, of companies that consider the Digital Twin as part of their future corporate strategy (Baskaran et al. 2019; Becue et al. 2018). Mainly, that is due to the constant development of information and communication technologies, which render the collection, storage, and analysis of ever-expanding amounts of data increasingly important (Uhlemann et al. 2017; Um et al. 2017). A Digital Twin serves as a useful concept to tackle data disruption between distributed systems and therefore represents a valuable contribution to improving business processes (Wang and Wang 2019). It allows for a complete semantic description of an asset, as it combines information with meta-information (Rosen et al. 2015). The literature corpus shows that the concept of the Digital Twin is incredibly valuable in manufacturing industries (Enders and Hoßbach 2019). Within the field of manufacturing, it is a declared aim for all assets to be able to connect both horizontally and vertically (Seif et al. 2019; Weber et al. 2017). Here, vertical integration refers to information technology systems of different hierarchical levels of a company, whereas horizontal integration describes the consideration and use of technologies between various actors and companies (Posada et al. 2015). The Digital Twin offers multiple opportunities to increase the effectiveness and productivity of production systems and is considered a suitable approach towards smart manufacturing (Wagner et al. 2019; Zhao et al. 2019b). In general and regardless of the respective field of application, Digital Twins offer further advantages, which mainly manifest themselves in an extensive aggregation of data that, extended by a semantic description, provide the basis for detailed analysis and simulations (Urbina Coronado et al. 2018).

With the adoption of Digital Twins, detached from applications such as simulation or machine learning, companies can generate significant added value from their data. Digital Twins enable data exchange between multiple stakeholders; their application is not limited to internal company processes and represents a suitable instrument for cross-company collaboration (Schleich et al. 2018; Schroeder et al. 2016; Wagner et al. 2017). In this context, Capiello et al. (2020) focus on the importance of Digital Twins in manufacturing and supply networks and emphasize the need for Digital Twins to allow for data sharing among network partners. Capiello et al. (2020) and Haße et al. (2020) introduce the term *Shared Digital Twin*, which extends the fundamental Digital Twin concept of describing information with meta-information to include the aspect of multilateral data sharing. The importance of Digital Twins, especially in a cross-company context, is increasing continuously. A study by Detecon shows that 80 percent of the future applications of Digital Twins in a 5 year time horizon will relate to cross-company usage aspects (Weber and Grosser 2019). To this end, Capiello et al. (2020) formulate several research questions on Shared Digital Twins in the context of Information Systems (IS) research, a fact that calls for the development of prescriptive design knowledge. A suitable medium for codifying and formulating prescriptive design knowledge are design principles (Chandra et al. 2015; Sein et al. 2011).

This research aims to develop design principles for *Shared Digital Twins*, which assist practitioners with guidance in their design and elevate case-specific design knowledge to enrich the knowledge base. For this purpose, the authors report on a qualitative interview study with 18 industry experts covering a broad industrial spectrum and representing all potential participants in a value chain. With these interviews, the study offers deep insights into the industry's current perspectives for and approaches to Digital Twins and is an opportunity to collect and formalize data on designing Shared Digital Twins. Accordingly, the article addresses the following research question:

**Research Question (RQ):** How to design Shared Digital Twins for inter-organizational data exchange?

Our study follows an aggregative logic, as we collect statements from the industry experts using a semi-structured interview guideline. The 18 interview experts provide statements about the requirements for Digital Twins, explicitly in the environment of cross-company collaborative utilization. These statements represent the meta-requirements (i.e., requirements addressing a class of artifacts (Walls et al. 1992)), forming the basis for the derivation of key requirements leading to the final development of design principles (Walls et al. 1992). Design principles are especially suitable for this purpose, as they are means to communicate research findings prescriptively both for researchers and managers (Hevner et al. 2004; Seidel et al. 2017). Design principles are an established instrument for disseminating knowledge in the context of Design Science Research (DSR) (Simon 1996). A chief purpose of design principles is their reusability beyond the borders of the application scenario from which they originate (Chandra Kruse and Seidel 2017; Iivari et al. 2020). If that were not true, design principles would lose their “(…) practical ethos” (Iivari et al. 2018, p. 1). To ensure for reusability, the study includes a second round of interviews with three industry experts in order to evaluate the design principles according to the recommendations of Iivari et al. (2020).

The paper is structured as follows. After the introduction, we present the theoretical background of Digital Twins in general as well as that of *Shared Digital Twins*. In this context, we introduce the use case Collaborative Condition Monitoring of the German Initiative *Plattform Industrie* 4.0 to illustrate the results elaborated within this contribution based on a practical example. The Sect. 3 follows, which outlines and details the design and execution of the qualitative interview study. This use case provides an essential, practice-related starting point that illustrates the importance of using Shared Digital Twins in distributed systems. Digital Twins enable holistic monitoring of machine conditions (see Enders and Hoßbach 2019 or van der Valk et al. 2021), therefore their use within a distributed system has a far-reaching impact on the collaborative utilization of technical operating data. In a further step, Sect. 4 presents the necessary key requirements, which as aggregated statements of the interview partners serve to derive the design principles for Shared Digital Twins in Sect. 5. This section deals with the developed design principles, technical implications, and exemplary quotations from expert interviews. Furthermore, Sect. 6 evaluates the design principles regarding their reusability. Section 7 presents the results as well as further implications and finally points out future research prospects.

## Theoretical Background

### Digital Twin Foundations

The first approach involving a Digital Twin originates from NASA’s early Apollo space projects. Within these projects, an identical version of the space capsule remained on Earth for test purposes during the mission (Rosen et al. 2015). However, this is not a Digital Twin by today’s understanding, as it lacks a virtual representation. The first mention of a Digital Twin by Professor Michael Grieves stems from the year 2003, who introduced the concept of the Digital Twin in a lecture on Product Lifecycle Management (PLM) (Grieves 2014). Therefore, Digital Twins have essentially emerged from PLM according to the original understanding. The most frequently cited definition to date is provided by Glaessgen and Stargel (2012). It describes the Digital Twin as "[…] *an integrated multiphysical, multi-scale, probabilistic simulation of a vehicle or system in its current form, using the best available physical models, sensor updates, fleet history, *etc*. to reflect the lifetime of the corresponding flying twin*" (Glaessgen and Stargel 2012, p. 7; Karakra et al. 2019). This definition includes essential aspects that characterize a Digital Twin. However, it is necessary to examine Digital Twins in more detail to enable a holistic investigation.

**Fig. 1:**
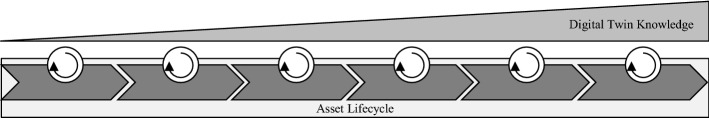
Digital Twin throughout the asset lifecycle according to Wang and Wang (2019)

The Digital Twin concept primarily focuses on providing data in a structured and semantically described form over the lifecycle of an asset (Glaessgen and Stargel 2012). The knowledge of the Digital Twin expands with the corresponding life cycle of the respective asset and thus, ideally, has a complete digital representation of all sensor and operating data, master data, and all relevant documents and CAD models (see Fig. 1). That distinguishes the Digital Twin concept from comparable approaches in the field of Big Data applications, such as Data Lakes or Data Warehouses. A data lake is “a large, raw data repository that stores and manages all company data bearing any format” (Sawadogo and Darmont 2021, p. 98). In contrast, a Data Warehouse “is generally understood as an integrated and time-varying collection of data primarily used in strategic decision making […] It is essentially a database that stores integrated, often historical, and aggregated information extracted from multiple, heterogeneous, autonomous, and distributed information sources.” (Hüsemann et al. 2000, p. 1). A Digital Twin combines both concepts as a data repository and uses these as an additional data source (Al-Ali et al. 2020). In contrast to previous database systems, users of Digital Twin have the advantage of possessing direct access to all relevant data of a specific asset without the need to search intensively. That leads to significant benefits such as reduced costs, innovation promotion, higher reliability, and easier decision-making (Jones et al. 2020). In this context, the taxonomy of Digital Twins by van der Valk et al. (2020) provides a comprehensive overview of the fundamental conceptual building blocks of Digital Twins. It bases on the analysis of 233 publications and illustrates eight essential dimensions and 18 characteristics of a Digital Twin (see Table 1). The following section bases on van der Valk et al. (2020).

**Table 1:**
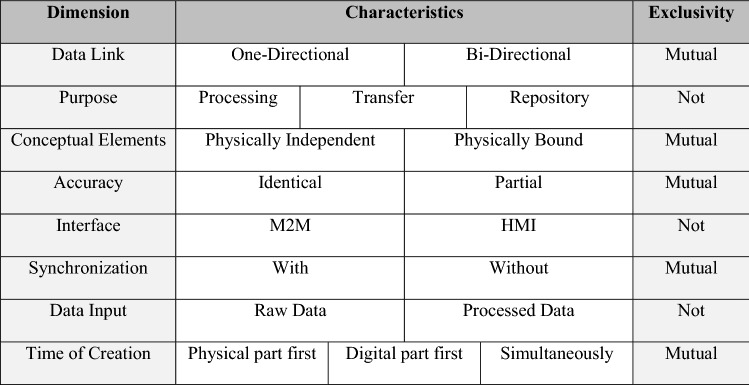
Taxonomy of Digital Twins (van der Valk et al. 2020)

The dimension *Data Link* is divided into the characteristics one-directional and bi-directional and describes the data flow between the Digital Twin and its physical counterpart. The dimension *Purpose* includes the characteristics of processing, transfer, and repository and defines the primary functions of a Digital Twin. *Conceptual Elements* is another dimension of Digital Twins and refers to the connection between the Digital Twin and its physical counterpart, either physically independent or physically bound. The dimension *Accuracy* specifies the level of detail of the Digital Twin concerning the respective physical counterpart and consists of the characteristics identical and partial. The dimension *Interface* consists of M2M and HMI characteristics and refers to the form of data transmission of a Digital Twin. *Synchronization* distinguishes between the characteristics with and without synchronization and describes the chronological alignment between the Digital Twin and the physical counterpart. The dimension *Data Input* outlines the various data formats that a Digital Twin must integrate and process and is therefore divided into raw and processed data. The dimension *Time of Creation* addresses the time of the actual instantiation of the Digital Twin in relation to the corresponding physical counterpart and consists of the three characteristics: physical part first, digital part first, and both simultaneously. Here, the individual characteristics are either mutually exclusive or non- exclusive.

The taxonomy shown in Table 1 provides the conceptual basis for developing the design principles for Shared Digital Twins, as it allows for a structured mapping of the principles identified. Furthermore, in the further course of this publication, it needs to be clarified whether the mentioned dimensions and characteristics of the taxonomy sufficiently represent the concept of the Shared Digital Twin. An important basic idea regarding the utilization of Digital Twins is an accurate mapping and description of processes and operations from the actual operating world (Weber and Grosser 2019). Due to this conceptual feature, a Digital Twin makes it possible to provide these data as a gateway to other actors within a cross-company network. However, a vast majority of Digital Twin applications still remain within a company (Weber and Grosser 2019).

### Shared Digital Twins

According to Longo et al. (2019), Digital Twins are particularly suitable for cross-company use in horizontal integration to prevent information asymmetries along a value chain. However, the topic of Data Sharing via Digital Twins has received little academic attention. Compared to the steadily increasing number of Digital Twins publications, the description of a cross-company use of Digital Twins occupies only a small part of the research area (see Table 2). For example, Ramm et al. (2020) describe Digital Twins as central platforms for collaboration. Furthermore, Uhlenkamp et al. (2020) investigate different levels of cooperation enabled by a Digital Twin. They conclude that Digital Twins, especially in combination with platforms and cloud hosting, only reach their full potential through cross-company use. In this context, Wang and Wang (2019) address, among other aspects, individual life cycle phases of a Digital Twin. Compared to Ramm et al. (2020) and Uhlenkamp et al. (2020), Wang and Wang (2019) describe a different form of cross-company data exchange since they address the transfer of ownership associated with the various life cycle phases. Most of these publications focus only on conceptual ideas, without showing concrete design approaches for this instance of Digital Twins. Although these publications provide valuable insights and initial ideas for the collaborative use of Digital Twins, they provide descriptive knowledge. They do not yet offer any prescriptive design approaches. Besides, most contributions do not include perspectives and views from the industry.

**Table 2:** Existing publications regarding the cross-company utilization of Digital Twins

Authors	Content	Findings
Wang and Wang (2019)	Data exchange between the various actors within the individual lifecycle phases based on Digital Twins	Descriptive
Ramm et al. (2020)	Collaborative usage of Digital Twins in medium-sized mechanical and plant engineering companies	Descriptive
Uhlenkamp et al. (2020)	Utilization of Digital Twins based on different forms of cooperation	Descriptive

Based on these aspects, we consider a Shared Digital Twin to be a specific instance of a Digital Twin, enabling the sharing and integration of data in multilateral and cross-company networks. The aim is to share data from the individual lifecycle phases of an asset across company boundaries and, at the same time, to enrich the Shared Digital Twin with data from external organizations. Accordingly, in this case, the aim is to embed a Digital Twin in a distributed system. A distributed system is a “collection of autonomous computing elements that appears to its users as a single coherent system” (van Steen and Tanenbaum 2017, p. 2). In this context, distributed systems are characterized by the design goals of sharing resources, transparency, openness, and *scalability* (van Steen and Tanenbaum 2017).

**Fig. 2:**
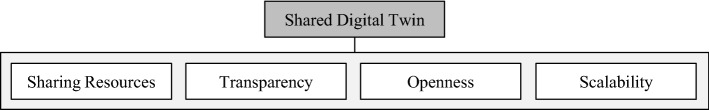
Design goals of Shared Digital Twins

These design goals directly impact the design of Digital Twins when deployed in a distributed system. That means that in *resource sharing*, the Shared Digital Twin must be able to provide submodels of the twin to the relevant participants. In the context of *transparency*, a Shared Digital Twin must supply the respective users with a complete overview of the shared submodel. Regarding *openness*, a Shared Digital Twin requires standardized subelements that enable the respective participants to access the elements of the Digital Twin without barriers. The *scalability* property of a Shared Digital Twin makes it possible to easily expand the number of potential participants. Therefore, the design goals of distributed systems have a direct impact on the design of Shared Digital Twins (see Fig. 2) and influence the design of the necessary functions that a Digital Twin must fulfill in a distributed system. In this context, Shared Digital Twins differ significantly from internally used Digital Twins and common inter-organizational information systems.

In relation to Digital Twins which are solely used internally, Shared Digital Twins differ in those requirements that arise due to the integration of various cross-company actors. The characteristics shown in the taxonomy primarily emphasize aspects that describe the relationship between the Digital Twin and the respective physical counterpart. Especially concerning the design goals shown in Fig. 2, Shared Digital Twins require greater consideration of interoperability and data security aspects. Both aspects are rarely considered in the context of the literature on Digital Twins and are at best described as a possible requirement (see Halenar et al. 2019, Jones et al. 2020 and Tao et al. 2019a). The use of Shared Digital Twins raises questions about data ownership in particular. Despite using the Digital Twin in a distributed system, the data owner must always be able to retain sovereignty over the data. These conceptual shortcomings of Digital Twins which are solely used internally need to be avoided and overcome when designing Shared Digital Twins.

Compared to standard inter-organizational information systems, Shared Digital Twins enable the collaborative use of the machine and operational data. In general, inter-organizational information systems are systems “that involve resources shared between two or more organizations” (Barrett and Konsynski 1982, p. 94). The data sharing described here goes beyond the standard data exchange in *Electronic Data Interchange (EDI)* and especially considers a collaborative aspect where, for example, new business models create additional values (Otto et al. 2019b). In the case of a Shared Digital Twin, this additional value is particularly evident in the fact that a holistic view of the asset over its entire lifecycle, including a complete semantic description of the data, is possible. Thus, it allows for the sharing of data that describes the behavior of an asset and therefore enables deep insights into its processes.

## Research Methodology

### Study Design

Our research generates design principles for Shared Digital Twins. As our data resides in the field, i.e., in the experiences of industry practitioners, we opted for a qualitative study with expert interviews. Qualitative studies are a common methodological approach when generating design principles before artifact instantiation (Möller et al. 2020b). Design principles, per se, are the formalization of prescriptive design knowledge (Chandra et al. 2015) and are a part of the theory of *design and action* (Gregor 2006). In that sense, design principles are not a traditional, material artifact (see March and Smith 1995), but an abstract meta-artifact that assists their users in designing artifacts more efficiently (Iivari 2003; Vaishnavi et al. 2004).

Our study consists of the four steps aggregated and proposed by Sarker and Sarker (2009). First, we started the study by identifying relevant industry experts from practice through immediate channels of senior personnel (similar to the ‚known sponsor approach ‘ (Patton 2002)). Weinstein (1993) distinguishes between epistemic and performative expertise. An expert with epistemic expertise is “a person who is capable of providing strong justifications for a range of claims in a domain” whereas a person with performative expertise “is able to perform a skill well according to the rules and virtues of a practice” (Weinstein 1993, p. 71). Within the scope of this study, we include experts with both epistemic and performative expertise. This results in a broad distribution of experts who have been responsible for or involved in both the actual implementation of Digital Twins and the planning and projecting of activities in this context. Furthermore, throughout the study, we used the snowball method or chain reference sampling, identifying additional suitable contacts for the study with help of the interviewees themselves (Biernacki and Waldorf 1981). To ensure a common understanding of relevant terminology, we asked a question regarding basic knowledge of Digital Twins at the beginning of each interview, allowing for a better classification of the answers.

**Fig. 3:**
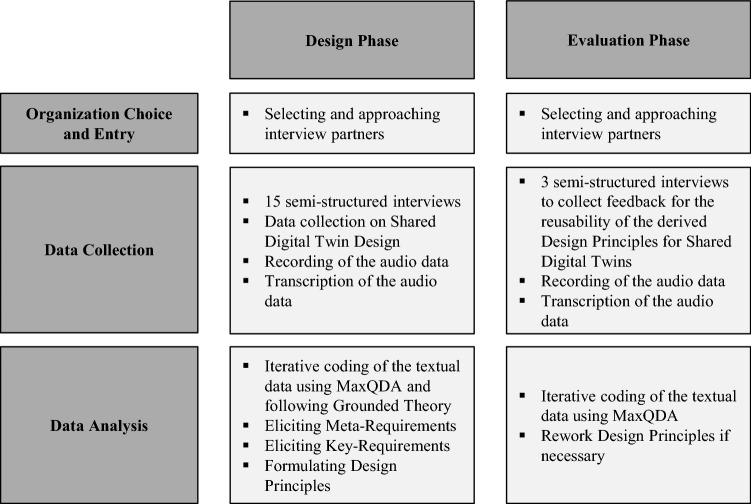
Methodological guidelines referring to Sarker and Sarker (2009)

We contacted each candidate with an invitation and a corresponding short presentation of the research project. Following the advice of Sarker and Sarker (2009) we retained flexibility and considered the interviewees’ restrictions for finding a suitable date. As the interview study was done during the COVID-19 global pandemic, all interviews were held remotely. The interviews were always conducted with a colleague so that there were always two interviewers and one interviewee in each interview. After we had collected the data, we followed the general principles of *Grounded Theory* to analyze the data qualitatively. As design principles are part of design theory and *Grounded Theory*, explicitly, it is a methodological approach to generate theory from all types of data. Thus, after formulating the design principles for Shared Digital Twins, we followed the recommendations of Iivari et al. (2020) and conducted interviews for evaluation with a smaller set of experts to validate our findings and stress test them for theoretical saturation. The aspects mentioned here are summarised in Fig. 3.

### Data Collection

We opted to split data collection dichotomously into two phases. The first phase collected data that we used to generate the results, i.e., the design principles. That phase entailed a significant body of expert interviews (*n* = 15). We interviewed another set of experts (*n* = 3) in the second phase to evaluate our findings.

The interviews were designed as *semi-structured* interviews with open questions to enable the experts to express their opinions freely. We chose a semi-structured interview as it provides a checklist of content that one needs to address to make the interviews comparable, yet, it also leaves enough freedom to react flexibly depending on the interviewees' answers (Merton and Kendall 1946; Myers and Newman 2007; Patton 2002). The interview guide is structured threefold. The first set of questions was meant to break the ice between interviewers and interviewees by asking personal questions about their backgrounds (Myers and Newman 2007). Next, the second set of questions aimed at uncovering requirements, principles, and general information on collaborative Digital Twin implementation. In that regard, the questions drew from the taxonomy of Digital Twins as proposed by van der Valk et al. (2020) and used the design dimensions as conceptual borders and as flexible starting points for inquiry. Lastly, concluding questions left the interviewee with the possibility to add any issues that might not have been addressed and gave space to comment on any topic freely. The interview guide itself was designed within the research group and created in multiple iterations.

We collected data for about four weeks with 15 industry experts to generate our initial design principles. Additionally, we had three interviews with experts in the second data collection period to evaluate our findings. The interviews lasted between 42 and 66 min for the design phase and between 39 and 51 min for the evaluation phase, with an average duration of 52 and 43 min. The notion of Digital Twins is strongly driven by industry practice. In this context, Table 3 shows the composition of the interview partners in terms of their positions, industry sectors, and the respective interview durations. Naturally, all interviewees were guaranteed to stay anonymous.

**Table 3:** Expert Interviews by position, sector, duration, and research phase

#	Position	Sector	Duration	Phase
1	Head of master data management	Mechanical engineering	42:38	Design phase
2	Global innovation manager	Logistics	49:00	
3	Enterprise architect	Electrical engineering	48:26	
4	Technology director	Electrical engineering	55:11	
5	Enterprise architect	Electrical engineering	53:42	
6	Production management	Mechanical engineering	45:36	
7	Senior research scientist	Electrical engineering	55:43	
8	Enterprise architect	Telecommunication	01:02:00	
9	Organizational development	Network operator	52:00	
10	Product line manager	Electrical engineering	54:41	
11	Quality manager	Electrical engineering	55:45	
12	Head of production technology	Mechanical engineering	01:06:14	
13	Innovation manager	Logistics	46:55	
14	Product management	Electrical engineering	50:52	
15	Head of data management	Chemical engineering	48:05	
16	Product management	Healthcare engineering	51:05	Evaluation phase
17	Head of R&D	Healthcare engineering	39:20	
18	Head of strategic management	Logistics	40:51	

As Digital Twins are an essential object of manufacturing analysis, we selected most interviewees from mechanical and electrical engineering (Enders and Hoßbach 2019). We aimed for a well-balanced distribution of interview partners across industries to mitigate industry biases, including logistics, telecommunication, and chemical industry experts. The main aim was not to limit the data collection to a specific industry and to select interview partners who were involved in digital transformation processes or had decision-making powers on the use of new digital technologies.

### Data Analysis

We transcribed the audio data fully, intending to leverage the maximum engraved information, which would hardly be possible by relying only on notes (Lapadat and Lindsay 1999). Also, transcribing is the first step when going in-depth into the data (Ochs 1979). Naturally, having the complete textual documentation of each interview makes the process of *coding* the data, i.e., attaching descriptive labels to portions of the data, more meaningful (Saldaña 2013). We used the software tool for qualitative data analysis MaxQDA both for transcription and coding.

The division of the contents along the four thematic blocks of the interview guideline allows a rough first structuring of the relevant contents. Nevertheless, the interview partners may already anticipate the contents of a subsequent thematic block. Therefore, coding is of importance in the context of data evaluation. Regarding the derivation of design principles for Shared Digital Twins, content-coding primarily involves identifying dimensions and characteristics beyond purely internally used Digital Twins. Especially in IS-Research, Grounded Theory often describes new technologies in emerging research areas (Birks et al. 2008; Urquhart and Fernandez 2006; Wiesche et al. 2017).

In the course of the evaluation, the authors decided to use selective coding. This type of coding differs slightly from the usual Grounded Theory methods, as the researcher defines some codes in advance to data analysis (Blair 2015). In the context of this research project, *template coding* is particularly well suited because the coding adapts to the dimensions of the taxonomy of Digital Twins by van der Valk et al. (2020). In the sense of Urquhart and Fernandez (2006), the authors do not consider the use of template coding as a deviation from pure Grounded Theory Methodology, but rather as an adaptation in the sense of a flexible approach that considers prior existing research.

### Design Principle Generation and Formulation

Design principles are constructed linguistically as prescriptive statements for action. They require structure in how they are formulated. The literature provides some templates for design principle formulation (Cronholm and Göbel 2018), (e.g., see Goldkuhl 2004 or van Aken 2004). We chose to adopt the template proposed by Chandra et al. (2015), as, next to linguistic guidance, it also gives underlying conceptual advice on building blocks of design principles. The template is as follows Chandra et al. (2015):

*“Provide the system with [material property – in terms of form and function] in order for users to [activity of user/group of users – in terms of action], given that [boundary conditions – user group’s characteristics or implementation settings].”*

Per se, design principles must address a class of artifacts rather than one instance (Sein et al. 2011). Thus, before formulating design principles, one must derive *meta-requirements* for an artifact that apply to a class of artifacts (Walls et al. 1992). The coding itself focuses on the taxonomy dimensions shown in Table 1 and follows *template coding*, meaning that some codes are already defined before the study (Blair 2015). At the same time, however, we paid attention to characteristics beyond these taxonomy dimensions extending the initial literature-based findings. Table 4 provides a sample of codes that are not part of the taxonomy but are nevertheless mentioned repeatedly by the interview partners. In this context, the use of the taxonomy forms the theoretical lens.

**Table 4:** Sample statements from the expert interviews including corresponding codes (see Saldaña 2013)

Data	Code
“Of course, we hope that this will lead to interoperability, i.e., the shared utilization of a Digital Twin. Of course, we also hope for business models based on a shared Digital Twin […].”	Interoperability
“Yes, of course, I think everyone should be the owner of the data that they distribute.”	Data security, data sovereignty

**Fig. 4:**
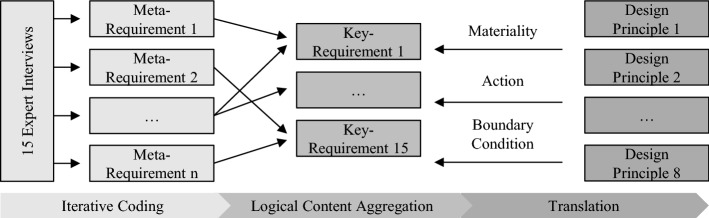
Design principle development according to Chandra et al. (2015), Möller et al. (2020a), and Walls et al. (1992)

To ensure that no design principle is without purpose, each design principle must address at least one *meta-requirement* (Goldkuhl 2004). Eliciting meta-requirements from interviews and generating responsive design principles that address them is a typical avenue for design principle generation (Möller et al. 2020b) (e.g., see Niemöller et al. 2019 or Feine et al. 2019). Following the advice of Koppenhagen et al. (2012), we applied *logical content aggregation* to synthesize meta-requirements to key-requirements to ensure that the resulting design principles actually address issues of high importance rather than a broad spectrum of particular problems (see Fig. 4). Additionally, that provides the benefit of limiting the number of design principles, which helps cognitive understandability (Miller 1956).

Following Pratt (2008) and the notion of ‘power quoting,’ we include illustrative excerpts from the interview transcripts throughout the presentation of the design principles to substantiate our findings. These power quotes are suppoed to be extraordinarily significant and catchy, to an extent that they cannot be paraphrased in a better way.

### Use Case – Collaborative Condition Monitoring

**Fig. 5:**
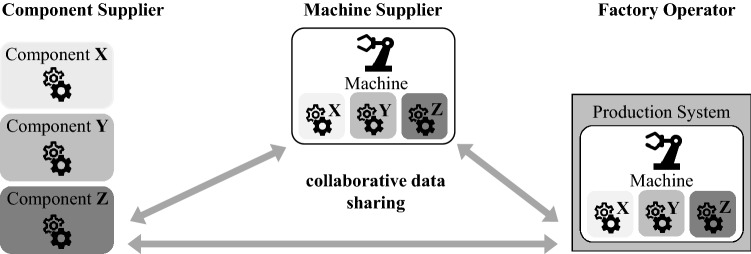
Basic structure of the Collaborative Condition Monitoring (Plattform Industrie 4.0 2020)

The *Collaborative Condition Monitoring* use case is based on a working group of the German Plattform Industrie 4.0 that develops collaborative, data-based business models. The foundation is a highly simplified, cross-company network consisting of a *component supplier*, a *machine supplier,* and a *factory operator* (see Fig. 5). The component supplier produces components for a machine, whereas the machine supplier assembles these components to construct a machine. The factory operator then uses this machine within a production system. The following points in this section relate to the publication Plattform Industrie 4.0 (2020).

A key aspect of the use case in this context is the collaborative use of technical operating data of a machine in a multilateral network. The technical basis here is formed by Digital Twins, provided along with the components. Furthermore, there is a Digital Twin of the entire machine which embeds the individual Digital Twins of the components. The factory operator is the data owner and can freely decide how to use the data. Therefore, the objective is to enable the factory operator to share the machine’s data with the component and machine supplier. The condition monitoring approach provides a rationale for this, whereby the factory operator receives additional services in return for providing the data, thus extending the machine's lifecycle. Another aspect here, however, is the conceptual design of the Digital Twins so that the operating data can be shared without barriers on the one hand and without compromising data sovereignty on the other. The design principles for Shared Digital Twins developed here provide an important entry point for the implementation of such a collaborative use case.

## Key Requirements for Shared Digital Twins

Table 5 shows the *key requirements* for Shared Digital Twins derived from the expert interviews, with the taxonomy of Digital Twins developed in van der Valk et al. (2020) serving as a framework to organize them, ontologically, since it allows a holistic view on the topic of Digital Twins. To do so, we use the 18 characteristics manifested within the eight dimensions *data link*, *purpose*, *conceptual elements*, *accuracy*, *interface*, *synchronization*, *data input,* and finally, *time of creation* (see Table 1).

**Table 5:** Short Description of the various Key Requirements

	Key requirements (KR)	Short description
Data link	Bi-directional data link (KR 1)	Refers to the communication between the physical and the virtual part and describes a simultaneous data flow between them
Purpose	Data processing (KR 2)	Refers to the functionality of a Digital Twin to further process incoming data sets, allowing for more detailed information regarding the counterpart
	Data repository (KR 3)	Refers to the capability to use the Digital Twin as a repository for the entire lifecycle
	Data transfer (KR 4)	Refers to the ability to transfer data from the Digital Twin to other systems or databases. This includes the data transfer from one Digital Twin to another
Interface	M2M (KR 5)	Refers to the direct communication between the Digital Twin and other devices in order to exchange data without any human interaction
	HMI (KR 6)	Refers to the capability of human interaction for monitoring purposes or system intervention
Synchronisation	On-demand data synchronization (KR 7)	Refers to the update of the Digital Twin being either in real time or non-real time
Data input	Raw data (KR 8)	Refers to the ability of a Digital Twin to capture and store unprocessed raw data such as sensor data
	Processed data (KR 9)	Refers to the ability of a Digital Twin to capture and store processed data
Data acquisition	Automated (KR 10)	Refers to a fully automated data input without any human intervention
	Semi-manual (KR 11)	Refers to a partially automated data input, which also includes human intervention
	Manual (KR 12)	Refers to a fully manual data input without any automated processes
Interoperability	Interoprable via interface (KR 13)	Refers to Digital Twins whose semantic models differ and where the input data is translated via the interface
	Entirely interoprable (KR 14)	Refers to Digital Twins whose semantic models are completely identical without the need for any translation
Data security	Usage control (KR 15)	Refers to multi-sided platforms allowing for sovereign data sharing in distributed networks, by adding policies to the data being shared

The dimensions *conceptual elements*, a*ccuracy,* and *time of creation* remain unconsidered, as they do not carry any relevant implications for a Shared Digital Twin in the case of *conceptual elements* and *accuracy* or, as in the case of the dimension *time of creation*, do not provide a basis for the derivation of a technology-oriented design principle. The three dimensions mentioned form the conceptual framework of a Digital Twin and show fundamental conceptual aspects of this concept. The dimension *conceptual elements* describes the constancy of the connection between the Digital Twin and its physical counterpart. In the use case presented in Sect. 3.5, a constant connection between the Digital Twin and the machine components is already under consideration. In terms of *accuracy*, a Digital Twin can only map components with a suitable sensor system, meaning that a completely identical digital mapping is impossible. In the case of *time of creation*, the Digital Twin emerges with the machine's components and starts integrating the data at the time of commissioning.

The dimension *data acquisition* is also relevant, although it is not represented in the original version of the taxonomy. Van der Valk et al. (2020) argue in their contribution: „*At first, we distinguished between an automated and a manual data acquisition but during the analysis, it became apparent that nearly all Digital Twins contain an automated data acquisition*.” However, in the wake of the expert interviews, this specific dimension turns out to be of relevance for the use of Digital Twins in distributed networks, which will be described in the further course of this contribution. Also, according to the experts, there are the dimensions *interoperability* and *data security*, as these are decisive features for a Digital Twin used for cross-company data sharing.

Table 5 lists the key requirements that aggregate logically connected meta-requirements. As per the wide variety of meta-requirements that emerged from the study, the key requirements only reflect those identified as being of decisive importance regarding the design of Shared Digital Twins. In conjunction with our findings, we define five mandatory characteristics that are so basic that they do not require instantiable, prescriptive guidelines but rather are obligatory components. In general, clearly identifying all relevant specifications is not entirely possible and requires a subjective evaluation by the coder (Glaser and Holton 2004). Overall, the coding is itself subject to an inevitable subjectivity (Blair 2015). To minimize possible inaccuracies, the present study's authors decided to derive these five mandatory characteristics to ensure the applicability of the design principles. These mandatory characteristics emerged from the first 15 expert interviews, where the interview partners emphasized characteristics that can be considered fundamental for instantiating a Shared Digital Twin. In contrast to the key requirement, the five mandatory characteristics are more general and represent a higher-level basis for cross-company data use and the requirements derived from this. Table 6 shows the five mandatory characteristics: ownership, data quality, data source, cybersecurity, and identification.

**Table 6:** Short description of the mandatory characteristics of Shared Digital Twins

Mandatory characteristics	Short description
Ownership	Before implementing a Shared Digital Twin, legal aspects must be clarified, in particular the terms of use and the ownership of the Shared Digital Twin
Data quality	When using a Shared Digital Twin, it has to be ensured that the data is suitable and valid for the application intended
Data source	A Shared Digital Twin must be able to obtain data from a variety of distributed and multi-organizational sources
Cyber security	A Shared Digital Twin must be protected against external attacks and manipulation to establish trust in the system
Identification	A Shared Digital Twin must be uniquely identifiable within the distributed network avoiding any confusion

## Design Principles for Shared Digital Twins

**Fig. 6:**
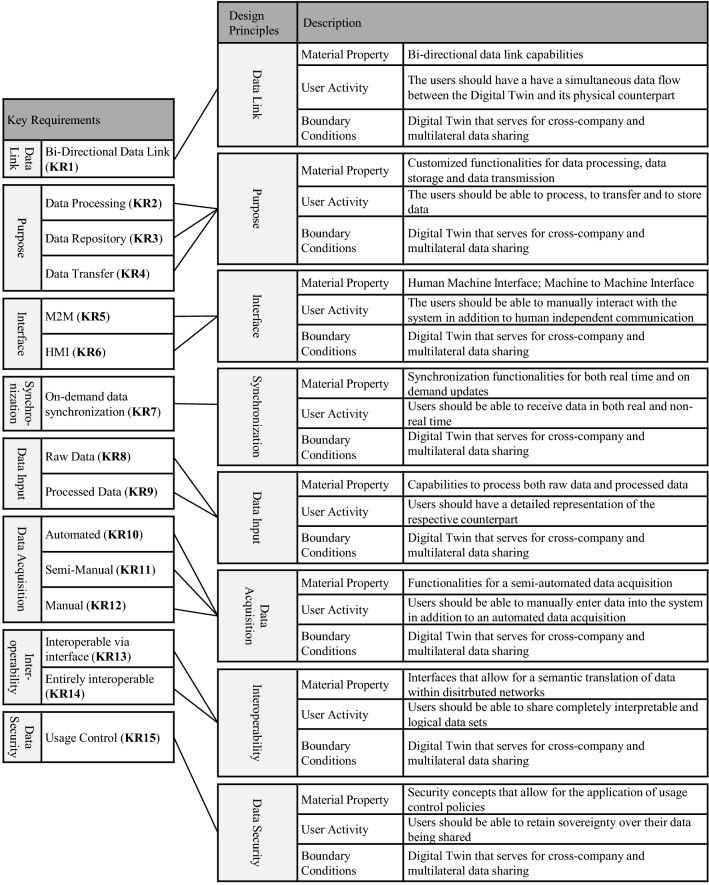
Design principles for Shared Digital Twins derived from expert interviews referring to Daiberl et al. (2019)

The design principles derived in this section serve as guidelines for developing Shared Digital Twins. For each design principle, the authors name possible technologies and concepts for implementation. Figure 6 shows the eight derived design principles in relation to the key requirements from Table 5. The dimensions and characteristics of Digital Twins described in the taxonomy (see Table 1) and the statements of the expert interviews serve as boundary conditions for the design principles developed here. According to Chandra et al. (2015), the relevant use context results from the application of Digital Twin data in distributed networks, where several users share a Digital Twin across multiple companies. The quotes shown here are based on a complete and detailed transcription of the interviews (King and Horrocks 2010). This procedure allows for a comprehensive overview of all interviewees’ statements so that the quotes chosen here are representative selections. Furthermore, we classify the individual characteristics within the use case Collaborative Condition Monitoring. This provides a better understanding of why the identified characteristics are relevant for the design of Shared Digital Twins.

### Data Link

*Design Principle 1*: Provide the Digital Twin with bi-directional data link capabilities in order for users to have a simultaneous data flow between the Digital Twin and its counterpart, given that the Digital Twin serves for cross-company and multilateral data sharing.

Illustrative Quote:

*“These are at least the questions we are asking ourselves, yes. Or have - exactly. Case by case it is different, that is. A data link for example. It can be one-directional, but it can also be bi-directional. It is not either or, but it is an and, often.”*

It is decisive for using a Shared Digital Twin to implement a one-directional data link between the Digital Twin and the physical counterpart. The link ensures an enrichment of the Digital Twin with the critical process parameters relevant for sharing. However, this one-directional data link is not enough in many cases, and it requires a bi-directional connection. Examples for this are use cases from the maintenance field where a plant operator provides the service company with access to the plant via the Digital Twin.

#### Use Case Illustration

Within the use case Collaborative Condition Monitoring, a bi-directional connection is crucial to allow data from component and machine suppliers to enter the machine via the Shared Digital Twin. Especially regarding extended service life, this feature is the only way to ensure that the data fed back leads to an adjustment of the machine parameters.

#### Technical Implication

In the case of a bi-directional connection between the physical and the virtual world, it is necessary to ensure that the counterpart has an interface capable of receiving control signals. This is initially less of a problem with newer machines since they already have a control logic that supports, e.g., the use of OPC-UA (Open Platform Communications Unified Architecture). In the case of older machines, this would require a retrofit solution that enables a corresponding communication. Overall, it is possible to integrate the logic of the field system directly into the Shared Digital Twin, allowing to control the commands directly via the Digital Twin. A bi-directional connection can have different characteristics. First, this can refer merely to the flow of information and knowledge back from the Digital Twin to the counterpart (Karakra et al. 2019). Alternatively, there is the possibility of a completely closed control loop, in which instructions from the Digital Twin flow back to the counterpart, leading to the control of an actuator (Kunath and Winkler 2018).

### Purpose

*Design Principle 2:* Provide the Digital Twin with customized functionalities in order for users to process, transfer and store data, given that the Digital Twin’s purpose is to enable cross-company and multilateral data sharing.

Illustrative Quotes:

*“I must be able to store it and I must be able to transfer it in any form, so that I can do meaningful processing.”*

*Purpose is also… Actually… Well, the primary topic is transfer, as I said, as an integration technology. But it is, in a sense also a repository and of course processing.”*

This design principle considers the possible applications of a Shared Digital Twin to the three characteristics of the dimension *purpose*. A Shared Digital Twin must fulfill all three dimensions to be used in a collaborative network. Both data in rest and data in use are crucial in this regard since a Shared Digital Twin stores all life cycle data of the counterpart on the one hand and shares the data with several stakeholders on the other. Furthermore, Shared Digital Twins must provide functionalities that allow for further processing of the stored data. These functionalities vary according to the individual requirements or use case and cover everything from simple monitoring tasks to machine learning applications or simulation.

#### Use Case Illustration

Especially in connection with the use case Collaborative Condition Monitoring, the three functions mentioned are of elementary importance, since this is the only possible way to achieve holistic collaboration via the Shared Digital Twin in the distributed system consisting of component and machine suppliers as well as factory operators.

#### Technical Implication

The implementation of additional functionalities that allow further data processing within the Digital Twin depends strongly on the characteristics of the individual use case. In principle, it is possible to integrate various functions for processing data as optional applications within Digital Twins, covering a wide range of possible purposes (Tao et al. 2018; Zheng et al. 2018). In data processing, the required functionality is strongly dependent on the respective application area and can, therefore, vary. However, it makes sense to consider functionalities such as simulations or monitoring applications in the form of add-on applications to be applied as required. There are also various data transfer options, whereas the Representational State Transfer (REST) is particularly suitable for distributed systems. Regarding the requirement that it should be possible to use a Shared Digital Twin as a data repository, there are approaches to creating a cloud platform for Digital Twins that enable different services to be provided via this platform (Borodulin et al. 2017). Within this platform, the data of the Digital Twin can be provided to various actors, who can perform further analyses based on it. Depending on requirements, the data itself can be stored via a *Hadoop file system* or in parallel via a combination of *SQL* and *NoSQL* databases (Al-Ali et al. 2020; Barth et al. 2020).

### Interface

*Design Principle* 3: Provide the Digital Twin with Interfaces in order for users to interact with the Digital Twin on the one hand and on the other to allow for a direct and human independent communication between distributed systems, given that the Digital Twin makes cross-company and multilateral data sharing possible.

Illustrative Quote:

*“I mean the interface both (M2M and HMI), yes, makes sense. So especially in this logistics-related world or where I really deal with physical entities, I think it makes sense to describe it that way, yes.”*

This design principle considers the need to allow for manual intervention in the processes. However, there is still the requirement to automate a significant part of the operations. The processes via the M2M interface operate autonomously, while an HMI interface provides the user with access to the process parameters of the counterpart.

#### Use Case Illustration

These aspects are relevant for Collaborative Condition Monitoring, making it possible to release submodels of the Shared Digital Twin for the relevant actors, especially via the HMI. These HMIs thus enable access to the respective released operating data as well as their analysis in order to improve the processes.

#### Technical Implication

The implementation of a Human–Machine Interface can be realized using various technologies. Examples are Virtual Reality (VR) applications or the implementation of a Graphical User Interface (GUI). VR applications allow for even more flexible interaction with the counterpart of the Digital Twin (Ma et al. 2019). Furthermore, a GUI offers the possibility to monitor all relevant process parameters for condition monitoring, for example (Haße et al. 2019). Moreover, it is possible to implement machine-to-machine communication via a REST API, transferring data to distributed systems.

### Synchronization

*Design Principle* 4*:* Provide the Digital Twin with convenient synchronization functionalities in order for users to receive both a constant real-time update of incoming data and on demand also a a non-real-time data update, given that the Digital Twin enables cross-company and multilateral data sharing.

Illustrative Quote:

*“So, I think there are many cases where the real-time connection is not crucial. And where on the other hand it would cost you a lot of money to implement. If I look at marketing and sales data now, sales data is nonsense, how my products are used by customers. I do an analysis every three seconds, I kind of do, I look at the product for a month and see how they have behaved, the twins that are outside, the physical twins how they have behaved this month, but I don't need the data from the last 4 milliseconds. On the contrary, I find it important to see where I need a real-time synchronization and where a discrete or sporadic synchronization is enough for me and makes it much easier for me to collect it.”*

This design principle specifies the data connection between the Digital Twin and its counterpart. Furthermore, this design principle describes the synchronization with distributed systems, where the Digital Twin receives data from external sources. The synchronization from distributed systems is very complex, so that a real-time update is not always possible. Moreover, a continuous real-time update requires considerable effort, which is often not appropriate for the respective use case. Therefore, the possibility of on-demand synchronization is important for a Shared Digital Twin. However, there are also use cases where real-time synchronization is of crucial importance. These include, for example, condition monitoring, where undesired process events must be detected immediately.

#### Use Case Illustration

In the context of Collaborative Condition Monitoring, it is important to transmit operating data in real-time. This enables the machine supplier, for example, to intervene in time in case of a sudden malfunction.

#### Technical Implication

Processing large amounts of data in real-time is particularly challenging. A Lambda architecture offers the possibility to process data both in real-time and on-demand, as it consists of two layers, each responsible for processing streaming and batch data (Gröger 2018). Such architectures are particularly suitable for monitoring processes, as they allow long-term analyses and rapid detection of process anomalies (Haße et al. 2019; Suthakar et al. 2019).

### Data Input

*Design Principle* 5: Provide the Digital Twin with capabilities to process both raw data and processed data in order for users to have a complete data set of the counterpart, given that the Digital Twin enables cross-company and multilateral data sharing.

Illustrative Quote:

*“Data input, I would expect, that it then flows from the Digital Twin into the companies. With raw data, processed data, that's really hard because you try to map it. […] I need raw data if I have a system somewhere that produces raw data. How do I feel me what temperature I have, it's more like raw data. With the other one (processed data) it is really a photographing data that is available somewhere.”*

This design principle refers to the type of data processed by a Digital Twin, considering both unprocessed raw data and already processed data. In a Shared Digital Twin, both aspects are important. Like an internally used Digital Twin, a Shared Digital Twin must process and store all raw data generated by the counterpart. To create an image of a counterpart that is as complete as possible, the Digital Twin is required to possess the ability to use various data sources and the ability to link together a number of other data formats. Many analyses require knowledge that the counterpart itself cannot generate.

#### Use Case Illustration

Especially when considering collaborative condition monitoring, both the consideration of raw and processed data is relevant. On the one hand, the respective component and machine suppliers need the raw data from the factory operator in order to gain insight into their use. On the other hand, the factory operator needs processed data in order to use it to improve processes.

#### Technical Implication

The processing of raw and processed data again requires additional functionalities in the form of applications selectable for the individual use case. Combining raw and processed data also requires the possibility to refine data through additional analyses. The integration of raw and processed data is closely related to the integration of structured and unstructured data. For a holistic representation of the individual lifecycle phases, it requires an architecture as described by Kassner et al. (2015). The integration layer delineated within this architecture allows for integrating different data sources and processing structured and unstructured data (Kassner et al. 2015).

### Data Acquisition

*Design Principle* 6: Provide the Digital Twin with functionalities to obtain a semi-automated data acquisition in order for users to still enter data manually if necessary, given that the Digital Twin enables cross-company and multilateral data sharing.

Illustrative Quote:

*“I mean, for example, in data acquisition. When I define a goal, for example in the cross-company area, I commit myself to semi-manual. This is already mentioned here that I say I also leave it manual, because otherwise I would exclude too many partners. They may not have the IoT for automated data acquisition.”*

This design principle refers to the possibility of manual data storage. The background of this design principle is an aspect of distributed networks, where not every participant has the technical prerequisite to enter data into the Digital Twin automatically. In order not to exclude individual partners, a Shared Digital Twin must be able to accept manually inserted data. Manual data acquisition is also crucial when it comes to the knowledge of domain experts. It must be possible to enter expert knowledge into the Digital Twin manually. Nevertheless, a Shared Digital Twin aims to automatically capture a large proportion of the data to keep operating effort as low as possible.

#### Use Case Illustration

Likewise, in connection with collaborative condition monitoring, the aim is to automate data acquisition as far as possible. Particularly regarding the use of operational data, the respective submodels of the Shared Digital Twin receive the respective data in an automated way. The use of a manual data transfer is particularly important for those component suppliers who cannot perform this automatically for technical reasons.

#### Technical Implication

Approaches for automated data acquisition are formed by IoT architectures, which have different elements, enabling connectivity to various systems. Examples of such architectures are the Siemens Industrial IoT operation system *Mindsphere* or *RIOTANA*, an architecture for real-time processing of raw sensor data (Haße et al. 2019; Jayanthi et al. 2019). In the context of data warehouses, the Extract, Transform, Load (ETL) process for merging data from multiple sources into a target system has proved to be a reliable tool for automated data acquisition (Machado et al. 2019; Trujillo and Luján-Mora 2003). Generally, ETL enables a systematic conversion of source data into the required format and combines it with a Lambda architecture (Galici et al. 2020). However, if an automated data integration is not applicable, it is possible to integrate the data into the Shared Digital Twin via a user interface field.

### Interoperability

*Design Principle* 7: Provide the Digital Twin with capable interfaces that allow for a semantic translation of the various data sets if the distributed Digital Twins are not fully interoperable in order for users to share completely interpretable and logical data sets, given that the Digital Twin enables cross-company and multilateral data sharing.

Illustrative Quotes:

*“So, there is non-interoperability, interoperability via interfaces and translators or full interoperability, because I can use common models [...] if I use a common model to create Digital Twins then I am fully interoperable, so to speak, because I follow a common standard. A Company “A“ language and a Company “B” language, they do not follow a common standard. That is why common standards can be so powerful.”*

*“On the technical side, I would then have to have exchange standards. The way we describe the Digital Twins at the moment and how we make them available - this is what is happening at our company. If another company uses a different solution, then they probably can't communicate with each other, which means that there would have to be some kind of standards for such an exchange.”*

This design principle is of crucial importance, especially for the aspect of multilateral data sharing. Shared Digital Twins must either be designed so that the Digital Twin and the distributed systems are fully interoperable or at least have interfaces that serve as translators. That applies to both the interfaces of the Shared Digital Twin and those of the distributed systems. Without this design principle, multilateral data sharing would not be possible; the exchanged data would not be interpretable and would have to be adapted to the data model of the respective system at great expense. Especially in terms of largely automated data acquisition, manual adaptation of incoming data is to be avoided.

#### Use Case Illustration

The dimension of interoperability is an important aspect, especially in the context of Collaborative Condition Monitoring, since data exchange within the collaborative network is only possible if all actors can use the semantic description of the operational data. The simplest solution would be for the individual actors to agree on using common semantic standards, but this is associated with obstacles, especially in the case of a larger consortium.

#### Technical Implication

In the context of this design principle, the technical implementation offers two possibilities. One possibility is that the participating companies commit themselves to a uniform data model, making the Shared Digital Twin fully interoperable with the respective distributed systems. An opportunity is offered by the German initiative Platform Industry 4.0 with its Asset Administration Shell constituing “*a knowledge structure that provides a description of the asset, its technical functionality and its relationship to other assets*” (Seif et al. 2019, p. 495). Accordingly, the AAS is a logical set of information with a complete semantic description of the respective counterpart (Wagner et al. 2017). Suppose the distributed systems and the Shared Digital Twin are not fully interoperable in their semantic data models. It is essential to have communication standards that enable the translation of the shared data for the target system. An example is the communication protocol OPC-UA, which serves as an interface between business applications and hardware and enables platform-independent interoperability (Katti et al. 2018).

### Data Security

*Design Principle* 8: Provide the Digital Twin with security concepts that allow for the application of usage control policies to enable the user to retain sovereignty over their data being shared, given that the Digital Twin enables cross-company and multilateral data sharing.

Illustrative Quotes:

*“My experience is that this is seen by many as extremely important. So respecting data ownership is essential. Especially nobody wants to give out any critical IP. […] So it is extremely important that the person who owns the product or who has done something with it, who has processed it, for example, that this information belongs to him or herself and that he or she can decide for himself or herself what information he or she wants to pass on to others.”*

*“[…] who is actually allowed to do what? What roles are there, who is allowed to do what to whom, who gets what data, that's what I would call data governance. At IDS, for example, the topic of data sovereignty, such roles are described.”*

In addition to design principle 7, this design principle is of decisive importance for a Shared Digital Twin in terms of data security. In general, all parties involved must be willing to share data, although there are numerous hesitations about sharing data. These hesitations must be overcome with the help of technical solutions. As with design principle 2 regarding the purpose of the Shared Digital Twin, the topic of data in use plays a decisive role here. The data provider must be able to determine what happens to the data after its release via the Shared Digital Twin. This approach goes beyond access control, which merely controls access by the data owner before release. A further aspect that has emerged from the interviews is the customer requirement for data sovereignty:

*“But this issue data sovereignty. That is simply a K.O. criterion for us, coming from the customer.”*

Often there are relationships between partners where the partners are equally dependent on each other's data. Both partners must ensure that they retain sovereignty over their data and that the shared data originate from trustworthy sources.

#### Use Case Illustration

Also, in the context of Collaborative Condition Monitoring, the individual actors must be able to retain sovereignty over their data. Particularly when releasing certain submodels of the Shared Digital Twin, the factory operator can attach terms of use to the data and thus restrict, for example, how long, how often, or how comprehensively the other actors can use the data. The restrictions depend on the exact terms of the business cooperation and still require legal coordination.

#### Technical Implication

The requirements mentioned in the interviews go beyond the restriction of access rights in the sense of attribute-based access control (ABAC) and relate more to the need to define rules for the use of data between the provider and the user. Usage control goes beyond traditional access control as it controls the future use of data and not only the regulation of access (Bussard et al. 2010). Another requirement for multilateral data sharing is data sovereignty, which is the ability of a natural and legal person to exercise exclusive self-determination over the economic asset data (Otto et al. 2019a). For the holistic realization of data sovereignty, the implementation of usage control policies is necessary (Zrenner et al. 2019). However, in the context of Digital Twins, we mainly find requirements which only define the restriction of access rights in terms of access control (Schleich et al. 2018; Steinmetz et al. 2018). Exceptions are Kern and Anderl (2020) who describe integrating an attribute-based usage control for Digital Twins. Aiming at data sovereignty in data ecosystems, the IDS initiative provides key concepts and technologies, allowing companies to share data with business partners while maintaining the right of self-determination over the data being shared (Otto et al. 2019a). Security gateways, according to DIN SPEC 27,070, referred to as IDS connectors, form standardized interfaces to receive, send, and transform data (Otto and Jarke 2019; Teuscher et al. 2020; Zrenner et al. 2019).

## Evaluation of the Design Principles

In additional interviews, the authors evaluate the compiled results from the first interviews and examine the eight design principles regarding their reusability. The basis for this is the questionnaire template for light evaluation of reusability of design principles, according to Iivari et al. (2020), which investigates questions regarding the reusability criteria accessibility, importance, novelty, and insightfulness, actability, and guidance, as well as effectiveness (Iivari et al. 2020). The questionnaire consists of 5 question blocks with the corresponding reusability aspects and a total of 19 questions. In principle, it is rather unusual to evaluate design principles. Nevertheless, Iivari et al. (2020) recommend “*to recruit at least a small “sample” of members of the target community for participating in the evaluation*” (Iivari et al. 2020, p. 23). The authors of this article follow this view and have evaluated the interviews with three additional experts (see Table 7).

**Table 7:** Exemplary expert quotes concerning the five reusability criteria of Iivari et al. (2020)

Reusability criteria	Exemplary quote
Accessibility	*“First of all, the principles are all understood, but of course they are very generic […] So I would say that this covers quite a lot.”*
Importance	*“What is of course important, as I understood it, is Design Principle 8: The security issue is becoming more and more important and I think indirectly, I have to talk about usage control policies, we notice this in the discussions, it's all about cyber and data security, how do we deal with it. Because these are the second and third questions that you hear from customers when it comes to this—what are we doing to encrypt our data accordingly.”*
Novelty and insightfulness	*“And when I think about it myself, I think it's absolutely valuable, absolutely helpful in setting up a Digital Twin in our company.”*
Actability and guidance	*“Basically, from my point of view this is understandable for me and I can imagine that you can get guidance with something like this. […] Well, I mean now, I can't exactly say that I have seen a limitation.”*
Effectiveness	*“We are talking about generic design principles and I would say that they are very helpful and if I ask myself now, if the design principles help to implement a Shared Digital Twin in reality, then I say: yes.”*

*Accessibility* describes the comprehensibility of the design principles for the target community. In this case, all experts claim to have understood the eight different design principles. In the context of the criterion *Importance*, experts highlight Design Principle 8 regarding the topic of data security in particular. However, in further discussions about the importance of the overall design principles, no expert questions them in their entirety. Regarding *Novelty and Insightfulness*, the experts particularly emphasize the aspect of cross-company use of Digital Twins and, at the same time, stress the importance of Shared Digital Twins for future applications. The experts rate the present design principles as very adequate, both in terms of *Actability and Guidance* and in terms of their *effectiveness*. Despite the generic description of the design principles, they provide sufficient implications for structuring implementation measures. As shown in Table 7, the design principle for data security is a fundamental criterion for Shared Digital Twins. Nevertheless, the evaluation refers to the entirety of all eight design principles. The evaluation conducted here is consistent with the approach of Iivari et al. (2020) and forms an important step in the development of usable design principles. Furthermore, this evaluation builds on established approaches in the DSR research area and includes explaining the goals, selecting a strategy for the evaluation, determining the properties to be evaluated, and designing individual evaluation episodes (Gregor et al. 2020; Venable et al. 2016).

## Conclusion, Limitations, Outlook

### Discussion

This paper aims to develop design principles for Shared Digital Twins. The use of Digital Twins offers considerable potential for cross-company use but requires a specific structure and implies special implementation requirements. This paper aims to identify these requirements, which must be reflected in this type of Digital Twins design. A significant added value of the present study lies in identifying characteristics that enable the use of Digital Twins in distributed systems. This identification goes beyond the examination of the corresponding literature and involves the expertise of professionals, allowing for increased consideration of relevant practical aspects.

**Fig. 7:**
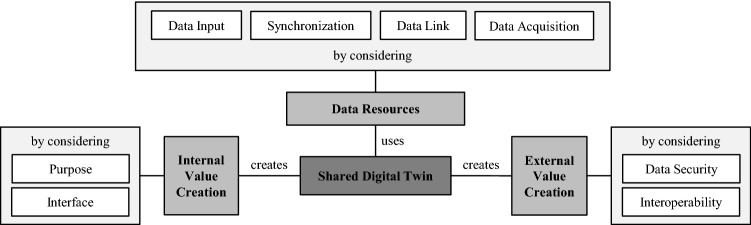
Ontology of design principles for Shared Digital Twins based on Barth et al. (2020)

In this context, the Digital Twin Conceptual Reference Framework by Barth et al. (2020) serves to classify the developed design principles. This classification highlights the conceptual difference to merely internally used Digital Twins. Therefore, we adapt the Reference Framework for Shared Digital Twins so that the ontology (see Fig. 7) allows a classification of the design principles into the sections *Internal Value Creation*, *External Value Creation,* and *Data Resources*. Using the ontology and the three classifications mentioned above, it is possible to show how the results of this study relate to each other and how the concept of the Shared Digital Twin differs from internally used approaches. First, the conceptual overlap between regular Digital Twins and Shared Digital Twins becomes apparent in the present review. Both approaches form a technology that can integrate data from multiple sources, describe them semantically and represent them over an entire lifecycle. However, the Shared Digital Twin goes much further in this respect in that it is technically capable of transferring the integration process to distributed systems. This emerges clearly from the *External Value Creation* feature, as Shared Digital Twins focus on interoperability and security features. In this context, *External Value Creation* describes the possibility of creating value based on cooperation with external actors (Barth et al. 2020), including Interoperability and Data Security principles. *Internal Value Creation* involves value creation achieved with processes within the company itself. This includes the Purpose and Interface specifications. In this case, *Data Resources* describe the design principles that enable the comprehensive recording of data, consisting of Data Input, Synchronization, Data Link, and Data Acquisition characteristics.

The use of Digital Twins in a distributed system extends previous approaches of an inter-organizational information system (Uhlenkamp et al. 2020). It forms a concept that significantly facilitates the collaborative use of operational and sensor data in a network of various actors. The data exchanged in this way allows detailed insights into operational processes and thus enables the emergence of new business models, for example, in maintenance (see Plattform Industrie 4.0 2020).

### Managerial Contributions

The derivation of the design principles based on 18 expert interviews with in-depth domain knowledge results in numerous managerial contributions, expressed in design principles 7 and 8 regarding interoperability and data security. Both design principles are key factors for participation in collaborative networks, enabling assets that have a Shared Digital Twin to share data with other stakeholders and use them for smart services as described by Azkan et al. (2020). Simultaneously, the design principles presented provide a practical guideline for implementing Shared Digital Twins, allowing practitioners to follow the eight categories of key requirements and the associated technical implications.

Again, the respective implementation steps depend largely on the context of the individual use case. It is often also a matter of identifying the appropriate software components that meet the requirements of the particular use case. The 15 key requirements from Table 5 serve as templates and provide a basis for workshops in which the respective focus groups can identify these software components. The use of Shared Digital Twins offers the opportunity to link the information flows of the individual stakeholders in a value chain more effectively. It thus creates the basis for developing business models based on the collaborative use of data. By considering the use case Collaborative Condition Monitoring, we also highlight the practical implications of the results described here. The collaborative use of operational data makes it possible for a network of component and machine suppliers as well as factory operators to gain insights into the use of components and the resulting improved service performance. Overall, this demonstrates a collaborative digital business model enabled by the use of a Shared Digital Twin, suggesting that the design principles developed here provide important added value for developing such collaborative business models. In this context, the application of Shared Digital Twins, as described within this contribution, offers the possibility of a holistic integration of operational data. Furthermore, the specifications of a Shared Digital Twin identified here provide information about the technical prerequisites for the collaborative sharing of technical, operational data. On the one hand, the use case benefits from these design principles, and, on the other hand, the use case itself illustrates the relevance of Shared Digital Twins by placing it into a practical context. Finally, the design principles we propose for Shared Digital Twins can very probably be applied to an array of similar use cases for Shared Digital Twins in distributed systems. Transferring the design principles might require adjustments in specific characteristics, for instance data security or interoperability, based on the specific requirements of different domains. However, they are a significant starting point to enable designers of Shared Digital Twins to draw from them in other cases and “(…) write their own versions of those principles (…).” (Chandra Kruse et al. 2016, p. 40).

### Research Contributions

This paper makes scientific contributions especially in expanding the literature-based knowledge to include the industry's requirements. Therefore, this paper essentially contributes to developing prescriptive knowledge for the design of Digital Twins used in collaborative networks. In addition to developing design principles for Shared Digital Twins based on 15 expert interviews, three other industry experts evaluate and rate these principles (Iivari et al. 2020). Design principles tend to be evaluated only rarely so that the present contribution addresses the demand for reusability of design principles.

Thus, this study differs from many other scientific contributions on the topic of Digital Twins, which mainly focuses on the manufacturing domain (Enders and Hoßbach 2019). Especially fundamental contributions such as those by van der Valk et al. (2020) or Jones et al. (2020) help to understand the basic characteristics of Digital Twins by providing descriptive knowledge. The aim basis of this paper is to extend this knowledge base to the collaborative use of Digital Twins and the enrichment of the existing descriptive knowledge with the practical knowledge of domain experts.

### Limitations

Regarding the limitations, we encountered the difficulty of keeping the design principles generic so that these principles address a class of artifacts and not only an instance (Sein et al. 2011). The focus in deriving these design principles is on technical design. Thus, not all aspects of expert interviews could be included. Legal issues, here classified as mandatory characteristics, also remain a realm which needs to be looked into more closely (see Table 6). Furthermore, this contribution illustrates that an entirely literature-based approach to developing descriptive knowledge in taxonomies is not sufficient to cover the topic of data sharing based on Digital Twins in a holistic manner. Especially the last aspect leads to the necessity to extend the taxonomy of van der Valk et al. (2020) with the aspects of a cross-company usage of Digital Twins in the course of further research. In this context, the dimensions of interoperability and data security must be mentioned again, which experts consider essential requirements for Shared Digital Twins. Accordingly, the topic of Shared Digital Twins must be examined from different perspectives that go beyond technical issues and have a legal and organizational context. This refers to standardization efforts that must be promoted during the cross-company use of Digital Twins and also refers to the examination of existing standards that can be taken into account.

### Outlook

The cross-company use of data is becoming increasingly important in industry and reveals the current barriers to sharing data with other companies. Key aspects of this are a lack of trust in the infrastructure and the fear of releasing critical data, thereby risking competitive disadvantages. In this context, the use of Shared Digital Twins provides the best solution as it relies on on existing structures and standards. In the future, concepts such as Shared Digital Twins will have to be accommodated in vendor-neutral integration platforms, which, on the one hand, enable the secure storage of data and, on the other hand, allow sovereign data sharing in the sense of collaborative use of data. Furthermore, it is worth to conduct further qualitative research to identify different configurations on the basis of various use cases. This could involve configurations that contain other characteristics from the taxonomy or provide for a different concept in terms of the level of data security. Another important aspect is instantiating a Shared Digital Twin based on the design principles identified here, which represents an part of our current research agenda.
